# A novel hybrid SCC*mec*-*mecC* region in *Staphylococcus sciuri*

**DOI:** 10.1093/jac/dkt452

**Published:** 2013-12-02

**Authors:** Ewan M. Harrison, Gavin K. Paterson, Matthew T. G. Holden, Xiaoliang Ba, Joana Rolo, Fiona J. E. Morgan, Bruno Pichon, Angela Kearns, Ruth N. Zadoks, Sharon J. Peacock, Julian Parkhill, Mark A. Holmes

**Affiliations:** 1 Department of Veterinary Medicine, University of Cambridge, Cambridge, UK; 2 Wellcome Trust Sanger Institute, Hinxton, UK; 3 Laboratory of Molecular Genetics, Instituto de Tecnologia Química e Biológica, Universidade Nova de Lisboa, Oeiras, Portugal; 4 Laboratory of Bacterial Evolution and Molecular Epidemiology, Instituto de Tecnologia Química e Biológica, Universidade Nova de Lisboa, Oeiras, Portugal; 5 Antibiotic Resistance and Healthcare Associated Infections Reference Unit, Public Health England, Colindale, London, UK; 6 Moredun Research Institute, Penicuik, UK; 7 Institute of Biodiversity, Animal Health and Comparative Medicine, University of Glasgow, Glasgow, UK; 8 Department of Clinical Medicine, University of Cambridge, Cambridge, UK

**Keywords:** β-lactams, MRSA, *mecA*

## Abstract

**Objectives:**

Methicillin resistance in *Staphylococcus* spp. results from the expression of an alternative penicillin-binding protein 2a (encoded by *mecA*) with a low affinity for β-lactam antibiotics. Recently, a novel variant of *mecA* known as *mecC* (formerly *mecA*_LGA251_) was identified in *Staphylococcus aureus* isolates from both humans and animals. In this study, we identified two *Staphylococcus sciuri* subsp. *carnaticus* isolates from bovine infections that harbour three different *mecA* homologues: *mecA*, *mecA1* and *mecC*.

**Methods:**

We subjected the two isolates to whole-genome sequencing to further understand the genetic context of the *mec*-containing region. We also used PCR and RT–PCR to investigate the excision and expression of the SCC*mec* element and *mec* genes, respectively.

**Results:**

Whole-genome sequencing revealed a novel hybrid SCC*mec* region at the *orfX* locus consisting of a class E *mec* complex (*mecI*-*mecR1*-*mecC1*-*blaZ*) located immediately downstream of a staphylococcal cassette chromosome *mec* (SCC*mec*) type VII element. A second SCC*mec att*L site (*att*L2), which was imperfect, was present downstream of the *mecC* region. PCR analysis of stationary-phase cultures showed that both the SCC*mec* type VII element and a hybrid SCC*mec*-*mecC* element were capable of excision from the genome and forming a circular intermediate. Transcriptional analysis showed that *mecC* and *mecA*, but not *mecA1*, were both expressed in liquid culture supplemented with oxacillin.

**Conclusions:**

Overall, this study further highlights that a range of staphylococcal species harbour the *mecC* gene and furthers the view that coagulase-negative staphylococci associated with animals may act as reservoirs of antibiotic resistance genes for more pathogenic staphylococcal species.

## Introduction

A wide range of staphylococcal species harbour the *mecA* gene encoding an alternative penicillin-binding protein 2a (PBP2a), which has a low affinity for β-lactam antibiotics and allows cell wall synthesis to occur in the presence of β-lactam antibiotics.^1–4^ *mecA*, along with its cognate regulators *mecI*-*mecR1*, are acquired as part of a larger mobile element known as staphylococcal cassette chromosome *mec* (SCC*mec*).^5^ SCC*mec* elements insert into the chromosome at the 3′ end of the *orfX* by site-specific recombination mediated by the CcrA and CcrB recombinases encoded on SCC*mec.*^6,7^ Coagulase-negative staphylococcal species are thought to be the source of *mecA* for methicillin-resistant *Staphylococcus aureus* (MRSA), with a number of studies having identified likely *in vivo* transfer events from a coagulase-negative staphylococcal species to *S. aureus.*^8–10^ The evolutionary origins of the *mecA* gene are thought to lie in the common ancestor of *Staphylococcus fleurettii*, *Staphylococcus vitulinus* and *Staphylococcus sciuri*,^11–13^ further supported by experimental evidence that the *mecA1* (*pbpD*) gene of *S. sciuri* is capable of mediating high-level β-lactam resistance in *S. aureus.*^13^

Recently, a novel allele of *mecA* was identified in MRSA from both humans and a range of animal species (livestock, small mammals and birds) across Europe.^14–19^ Further work in Denmark identified likely transmission events between livestock and humans, suggesting a zoonotic reservoir for the human isolates.^20,21^ This type of *mec* is named *mecC* (originally *mecA*_LGA251_) and shares 70% nucleotide identity with *mecA.*^18,22^ The *mecC* gene is present with its cognate regulators *mecI*-*mecR1* as part of a class E *mec* complex that shares structural similarity (*mecI*-*mecR1*-*mecC*-*blaZ*) with a *mec* gene complex found in *Macrococcus caseolyticus.*^23^ The class E complex is present as part of a larger 29.4 kb SCC*mec* type XI inserted at *orfX*, which also encodes the recombinase genes *ccrA*/*B* and arsenic resistance genes.^18^ We recently described an isolate of *Staphylococcus xylosus* with a novel allotype of *mecC* (*mecC1*) present as part of a possible ancestral SCC*mec* element.^24^ In this work, we describe two *S. sciuri* subsp. *carnaticus* isolates cultured from skin infection in cattle that harbour three distinct types of the *mec* gene (*mecC*, *mecA* and *mecA1*). This is the first demonstration of *mecC* in *S. sciuri* and suggests that, like the ‘conventional’ *mecA* gene, *mecC* is also present in a range of staphylococcal species found in animals. This isolate also carries a novel hybrid SCC*mec* consisting of SCC*mec* type VII, encoding *mecA* and a separate *mecC* region.

## Materials and methods

### Bacterial strains and growth conditions

Isolates were grown on blood agar (Oxoid, UK) and in tryptone soya broth (TSB) at 37°C. A list of isolates used in this study is shown in Table 1. Antimicrobial susceptibility testing was performed using disc susceptibility testing according to BSAC criteria (BSAC Methods for Antimicrobial Susceptibility Testing Version 11.1 May 2012). Isolates were tested for resistance to oxacillin, chloramphenicol, erythromycin, cefoxitin, ciprofloxacin, penicillin, neomycin, tetracycline, fusidic acid and gentamicin. NCTC 12493 and NCTC 6571 were used, respectively, as control resistant and susceptible isolates for oxacillin and cefoxitin.

**Table 1.** Isolates of *S. sciuri* subsp. *carnaticus* and key genotypic and phenotypic characteristics described in this study

Isolate	Resistance genotype^a^	Resistance phenotype^b^	Reference
GVGS2	*str*, *blaZ*, *mecA*, *mecC*, *mecA1*, *erm*(C), *fexA*, *tet*(K)	OXA, CEF, CHL, PEN, TET, FUS	this work
GVGS3	*str*, *blaZ*, *mecA*, *mecC*, *mecA1*, *fexA*, *tet*(K)	OXA, CEF, CHL, PEN, TET, FUS	this work

^a^*str*, streptomycin resistance; *blaZ*, β-lactamase (penicillin resistance); *mecA*, β-lactam resistance; *mecC*, β-lactam resistance; *mecA1*, potential for β-lactam resistance with a promoter mutation;^51^ *fexA*, chloramphenicol resistance; *tet*(K), tetracycline resistance; *erm*(C), erythromycin resistance.

^b^OXA, oxacillin; CEF, cefoxitin; CHL, chloramphenicol; PEN, penicillin; TET, tetracycline; FUS, fusidic acid.

### Whole-genome sequencing

Genomic DNA of *S. sciuri* isolates GVGS2 and GVGS3 was extracted from overnight cultures grown in TSB at 37°C using the MasterPure Gram Positive DNA Purification Kit (Cambio, UK) or by the isothiocyanate/guanidine method.^25^ Illumina library preparation was carried out as described by Quail *et al*.^26^ and Hi-Seq sequencing was carried out following the manufacturer's standard protocols (Illumina, Inc., USA).

### Sequence analysis and phylogenetics

Contigs for GVGS2 were assembled *de novo* from Fastqs with Velvet.^27^ Contigs containing the *orfX* region were closed by PCR using specific primers at the ends of each contig and ABI sequencing of the resulting PCR amplicons (Source Bioscience, Cambridge, UK). Sequences of the *orfX* region in *S. sciuri* isolate GVGS2 were submitted to the EMBL database under the accession number HG515014. Annotation was carried out using the automated RAST server^28^ and then manually with Artemis.^29^ Orthologous proteins were checked against the NCBI or EBI databases using BLAST. Comparative genomics was carried out using WebACT^30^ and viewed with the Artemis comparison tool (ACT).^31^ The presence of antibiotic resistance genes was identified using the ResFinder-1.3 Server (http://cge.cbs.dtu.dk/services/ResFinder/)^32^ and by BLAST. Nucleotide sequences of *mecA* homologues were aligned using ClustalW in Seaview^33^ and a maximum likelihood tree was generated using RAxML.^34^

### PCR for SCCmec excision

Primers were designed using Primer 3 (http://primer3.sourceforge.net). Genomic DNA was extracted using the MasterPure Gram Positive DNA Purification Kit (Cambio, UK) from stationary-phase cultures grown in TSB. PCR was carried out using MyTaq DNA Polymerase (Bioline, UK). Primer sequences are listed in Table 2. PCR amplicons were ABI sequenced (Source Bioscience, Cambridge, UK).

**Table 2.** Oligonucleotide primers used in this study

Primer name	Sequence 5′–3′	Target/function	Source
P1	TATCATCGGCGGATCAAACG	detection of SCC*mec* excision	this work
P2	TGCGGAGGCTAACTATGTCA	detection of SCC*mec* excision	this work
P3	TTGCCAATTAAAAGGTTGGTTAG	detection of SCC*mec* excision	this work
P4	TCTCAAGTAACATCTCAGCAATGA	detection of SCC*mec* excision	this work
P5	TGTGGTGCCAATGTCAAAGT	detection of SCC*mec* excision	this work
P6	TCGCTTTACAAGTGTCATGTTT	detection of SCC*mec* excision	this work
MecA1	GTAGAAATGACTGAACGTCCGATAA	*mecA*	^52^
MecA2	CCAATTCCACATTGTTTCGGTCTAA	*mecA*	^52^
mecC-Uni-F	GGATCTGGTACAGCATTACAACC	*mecC*/*mecC1*	this work
mecC-Uni-R	TGCTTTAAATCRATMTTGCCG	*mecC*/*mecC1*	this work
mecA1-spec-F	TTGAAGAAGCAACAACGCAC	*mecA1*	this work
mecA1-spec-R	GAACCGTAGTCATCTTTCATGTTG	*mecA1*	this work
Uni-16s-Ctrl-F	ACACGGTCCAGACTCCTACG	16S rDNA	this work
Uni-16s-Ctrl-R	ATAATTCCGGATAACGCTTGC	16S rDNA	this work

### Oligonucleotide primer design and strain screening

The sequences of *mecC* from *S. aureus* LGA251 and *S. sciuri* GVGS2 and *mecC1* from *S. xylosus* S04009 were aligned with Seaview^33^ and conserved primers were designed using Primaclade.^35^ The presence of *mecC* was confirmed by PCR on boilates or genomic DNA using primers: mecC-Uni-F and mecC-Uni-R. Primer sequences are listed in Table 2. Boilates were prepared by inoculating two or three single colonies in 50 μL of sterile H_2_O and boiling for 5 min, followed by centrifugation at 16 000 **g** for 2 min.

### Transcriptional analysis of mec gene expression by RT–PCR

Isolates GVGS2 and GVGS3 were grown in 5 mL of TSB supplemented with 0.1 mg/L oxacillin overnight at 37°C with 200 rpm shaking. After ∼16 h, the cultures were diluted 1/50 into 5 mL of fresh TSB supplemented with 0.1 mg/L oxacillin and grown for 3 h under the same conditions to an optical density of ∼0.8 at 595 nm. An *S. sciuri mecA*/*mecA1*-positive isolate and an ST130 *S. aureus* *mecC*-positive isolate were also grown under the same conditions as controls. Total RNA was then extracted from 1 mL of culture using the SV Total RNA Isolation System (Promega, UK) following the manufacturer's standard protocol for Gram-positive bacteria. After an additional DNAse step using RQ1 RNase-Free DNase (Promega, UK), cDNA was synthesized using ProtoScript^®^ II Reverse Transcriptase (NEB, UK) and a Random Hexamer primer (Fisher Scientific, UK) following the manufacturer's standard protocol. Controls without reverse transcriptase were generated for all samples and showed no amplification in the subsequent PCRs. cDNA was used undiluted in a standard PCR for the detection of *mecC* (mecC-Uni-F/R), *mecA* (MecA1/A2) and *mecA1* (mecA1-spec-F/R) (Table 2). PCR was carried out using MyTaq DNA Polymerase (Bioline, UK). A PCR for 16S rRNA (Uni-16s-Ctrl-F/R) was also carried out as a positive control for cDNA synthesis (Table 2).

## Results

### Multidrug-resistant S. sciuri subsp. carnaticus from wound infections in cattle

A farm in the south-west of England had multidrug-resistant bacterial infections in caesarean incision wounds in several Belgian Blue cattle. Multidrug-resistant *Staphylococcus* species (Table 1) were isolated from wound swabs taken from two cows (GVGS2 and GVGS3); both isolates were subjected to whole-genome sequencing. Analysis of 16S rRNA genes revealed these isolates to be *S. sciuri.* Further sub-speciation by BLAST comparison of the *hsp60*, *sodA*, *dnaJ* and *tuf* genes against the NCBI database identified the isolates as *S. sciuri* subsp. *carnaticus.*^36,37^ BLAST comparison of the four largest contigs (total size of contigs: 703 911 bp, ∼26% of GVGS2 genome) of the complete GVGS2 *de novo* genome assembly against GVGS3 identified only one single-nucleotide polymorphism (SNP), suggesting that the two isolates were very closely related (the same strain). The two isolates were resistant to a range of antimicrobial drugs (Table 1). Analysis of the genome sequence identified a number of resistance genes, including *str*, *erm*(C) (GVGS2 only), *fexA* and *tet*(K). These findings match the phenotype for these isolates, except for isolate GVGS2, which was susceptible to erythromycin on disc testing despite being positive for *erm*(C) (Table 1). Further analysis of the GVGS2 *erm*(C) gene revealed it to be part of a putative ∼2.5 kb plasmid (data not shown). The *erm*(C) gene was intact, but contained an Ile123Val substitution compared with the most closely related *S. aureus erm*(C) sequences in the NCBI database (accession number YP_001901404).

### The orfX region of isolate GVGS2 contains both mecA and mecC

**Figure 1.**
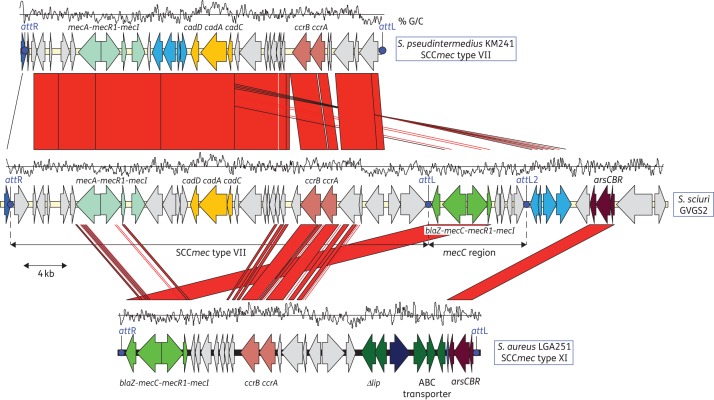
Comparison of the novel hybrid SCC*mec-mecC* in *S. sciuri* isolate GVGS2 (EMBL accession number HG515014), SCC*mec* type VII in *S. pseudintermedius* strain KM241 (EMBL accession number AM904731) and SCC*mec* type XI in *S. aureus* LGA251 (EMBL accession number FR821779). Areas of red show regions conserved between the two sequences and homologous coding DNA sequences are marked in the same colour. Blue dots indicate the SCC*mec att* sites. The percentage G/C content of the region is shown above each genome schematic.

**Figure 2.**
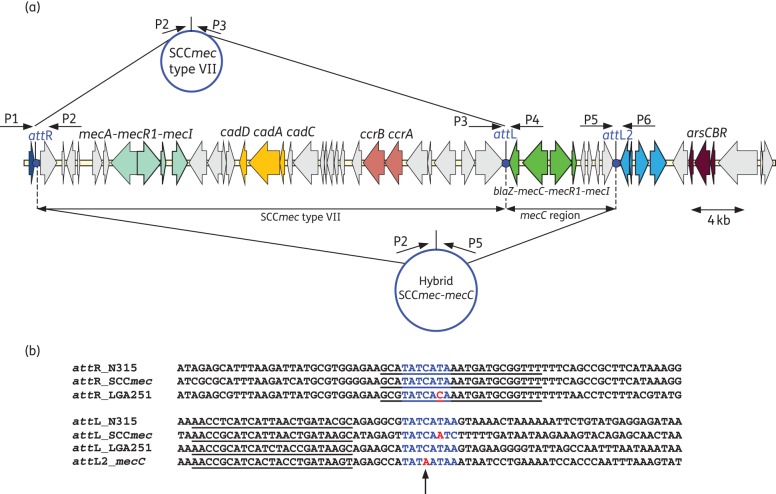
SCC*mec-mecC* element excision and repeats. (a) Schematic representation of potential excised circular SCC*mec* and hybrid SCC*mec*-*mecC*, and location of PCR primers used to detect excision. (b) DNA sequences of *att*R and *att*L sites in *S. aureus* N315 (N315), SCC*mec* type VII in *S. pseudintermedius* strain KM241 (SCC*mec*), SCC*mec* type XI in *S. aureus* LGA251 (LGA251) and downstream of the *mecC* region in *S. sciuri* GVGS2 (*mecC*). The bases that make up the inverted repeat are underlined. The bases in blue represent the core 8 bp regions identified in the *att*B site with mutations highlighted in red.^41^ The central cytosine is thought to be essential for *att*B × *att*SCC recombination and is highlighted with an arrow.^41^

BLAST analysis identified that both isolates (GVGS2 and GVGS3) harboured three different homologues of the *mecA* gene: *mecA*, *mecA1* and *mecC*. We further analysed the genome of GVGS2 in detail and identified that two of the *mecA* homologues (*mecA* and *mecC*) were found at the *orfX* locus (the SCC*mec* insertion site) (Figure 1), while *mecA1* was part of the previously reported chromosomal locus that shared the greatest similarity to *S. sciuri* subsp. *carnaticus* strain ATCC 700058 (accession number AB547236) (data not shown).^12^ Comparative genomics of the *orfX* locus identified that the region was made up of two distinct parts; immediately downstream of the *orfX* locus was an SCC*mec* element that is most closely related to the SCC*mec* type VII in *Staphylococcus pseudintermedius* strain KM241 (Figure 1).^38^ The SCC*mec* in GVGS2 differed from the SCC*mec* type VII in *S. pseudintermedius* by the presence of a number of extra genes and a small deletion. Firstly, an extra hypothetical protein and a putative short-chain dehydrogenase/reductase were present at the 5′ end proximal to *orfX* and downstream of the *ccrB*5 gene, respectively, both of which are absent in *S. pseudintermedius*. Next, the two small hypothetical proteins present upstream of the *ccrA* gene in the *S. pseudintermedius* SCC*mec* were absent in GVGS2. At the 3′ end of the SCC*mec*, an extra AAA superfamily ATPase and a putative serine protease were also present in *S. sciuri*. The SCC*mec* element was bounded by two intact repeats (SCC*mec att*R and *att*L) (Figures 1 and 2). The region containing the *mecC* gene was immediately downstream of the SCC*mec* element and was bounded by a second SCC*mec att*L site at the 3′ end (*att*L2) (Figures 1 and 2). The *mecC* gene, as in *S. aureus* and *S. xylosus*, was part of a homologous class E *mec* gene complex (*mecI*-*mecR1*-*mecC*-*blaZ*).^18,24^ The *mecC* gene in GVGS2 shared 96.3% nucleotide identity with *mecC* from LGA251 and 91% nucleotide identity with *mecC1* from *S. xylosus*. The other genes, *mecI*, *mecR1* and *blaZ*, shared 95.6%, 97.1% and 97.7% nucleotide identity, respectively, with their respective homologues in LGA251. Four other genes were present between the *mec* gene complex and *att*L2. Immediately downstream of *mecI* was an AsnC family transcriptional regulator and putative glyoxalase, which were most closely related to an AsnC family transcriptional regulator in *Clostridium arbusti* SL206 (accession number ZP_10773559) and a glyoxalase/bleomycin resistance protein in *Paenibacillus* sp. JDR-2 (accession number YP_003008991), respectively. Next, there was a PhnB-like protein and a DeoR family putative transcriptional regulator, which are found in a number of *S. aureus* SCC*mec* elements in the DDBJ/EMBL/GenBank databases. Immediately downstream of the *mecC* region *att*L2 were four genes also present in the full SCC*mec* VII element upstream: a putative transmembrane protein, a putative transcriptional regulator, a rhodanese domain-containing protein and a metallo-β-lactamase superfamily protein. These genes are also part of the *S. fleurettii* chromosomal *mecA* locus that has been suggested to be the template for the *mec* complex in *mecA* SCC*mec* elements.^12^ Finally, downstream of this was an arsenic resistance gene cluster, *arsCBR*, which is part of the SCC*mec* type XI and which was also found to be present in the chromosome of *S. xylosus* S04009.^24^

### Both the SCCmec type VII and a hybrid SCCmec-mecC can excise from the chromosome

Previously, excision of a tandem arginine catabolic mobile element (‘ACME’)-SCC*mec* and a SCC*mec* type IV cassette at a secondary *att*R site (*att*R2) was reported in *S. aureus.*^39,40^ Further analysis of the flanking repeats showed that the *att*L2 repeat downstream of the *mecC* region contained an SNP (C to A) at the central cytosine previously shown to be essential for recombination between *att*B and *att*S (*att*SCC), suggesting that this repeat might not be functional (Figure 2b).^41^ The *att*R of the SCC*mec* also contained an SNP in the central 8 bp region in comparison with the *att*R of *S. aureus* N315 (T to A); however, substitutions in this position have been demonstrated not to adversely affect recombination.^41^ Therefore, as the SCC*mec* and *mecC* region in GVGS2 are bounded by a single *att*R and two different *att*L sites (*att*L and *att*L2) (Figures 1 and 2a) we designed PCR primers in order to detect excision and circularization of either the SCC*mec* type VII element alone (*att*R × *att*L) or a putative larger hybrid SCC*mec*-*mecC* element (*att*R × *att*L2) (Figure 2a). PCRs were designed to amplify across the *orfX att*B region if excision of either the SCC*mec* type VII alone (P1 + P4) or a hybrid SCC*mec*-*mecC* (P1 + P6) element occurred. A second set of PCRs were carried out to detect the putative extrachromosomal circular forms of either the SCC*mec* type VII (P2 + P3) or the SCC*mec*-*mecC* (P2 + P5) hybrid (Figure 2a). PCR conducted on ∼250 ng of genomic DNA from stationary-phase cultures produced weak positive PCR amplicons for P1 + P4, P1 + P6, P2 + P3 and P2 + P5 primer combinations. Sequencing of the PCR amplicons confirmed formation of *att*B between both *att*R × *att*L (P1 + P4) and between *att*R × *att*L2 (P1 + P6). Sequencing also confirmed the formation of the *att*SCC (present in the circular form) between *att*R × *att*L of the SCC*mec* type VI (P2 + P3) and the *att*R × *att*L2 of the hybrid SCC*mec*-*mecC* (P2 + P5).

### Transcriptional analysis of mecC and mecA

In order to assess if both *mecC* and *mecA* were expressed in the same isolate, *S. sciuri* GVGS2 and GVGS3 were subjected to transcriptional analysis in the presence of low levels of oxacillin (0.1 mg/L). RT–PCR for *mecC* and *mecA* confirmed that both genes were expressed in GVGS2 and GVGS3 under the conditions tested, while no *mecA1* transcript was detected.

### Screening of S. sciuri isolates for mecC

Using a multiple sequence alignment of *mecC* from *S. aureus* LGA251, *S. xylosus* S04009 and *S. sciuri* GVGS2, we designed universal *mecC* primers and tested a selection of *S. sciuri* isolates to determine the prevalence of *mecC* genes. We tested 11 isolates of *S. sciuri* subsp. *carnaticus* isolated between 1990 and 1992 from different hosts (cattle, rodents and cetaceans) in the USA^42^ and 12 isolates from human clinical infections in England sent to Public Health England for further testing between 2006 and 2011. None of the isolates were positive by PCR for *mecC*.

## Discussion

**Figure 3.**
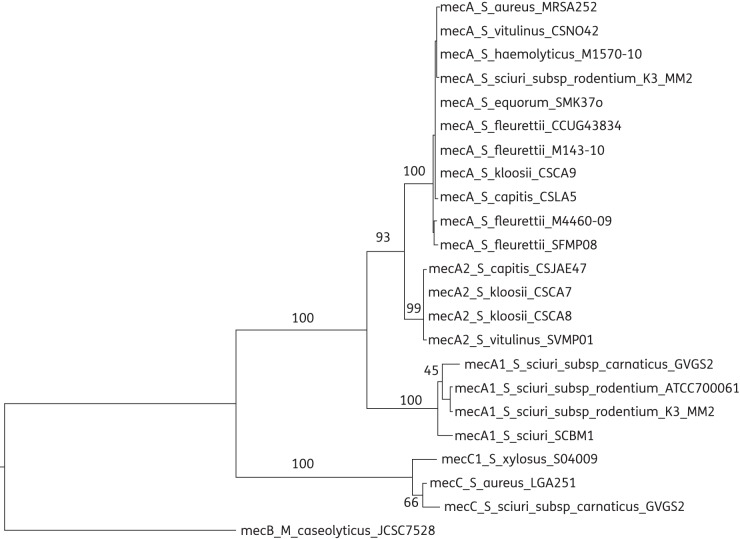
Phylogenetic relationships of *mec* homologues. Maximum likelihood tree of nucleotide sequence of *mec* homologues. The tree is rooted in *M. caseolyticus* *mecB* as an outgroup. Bootstrap values for branches are shown.

In this work, we have identified a further staphylococcal species that harbours the *mecC* gene. The *mecC* from GVGS2 is more closely related to *mecC* from *S. aureus* than *mecC1* from *S. xylosus*. Phylogenetic analysis of *mec* gene homologues shows that the *S. xylosus mecC1* probably represents a more ancestral form of *mecC*, as previously suggested (Figure 3).^24^ Like both *S. aureus* LGA251 and *S. xylosus* S04009, the *S. sciuri* isolates harbouring the *mecC* gene were again obtained from a bovine host, suggesting that selective pressure for the maintenance of *mecC* might be present in this or a closely linked ecological niche. *mecC* was also recently identified in a *Staphylococcus stepanovicii* isolate from a wild Eurasian lynx (*Lynx lynx*), suggesting that *mecC*-positive staphylococci are also present in diverse wildlife populations, as reported for *S. aureus mecC* isolates.^16,43,44^ We found that both *mecC* and *mecA* were expressed under laboratory growth conditions with low levels of oxacillin, suggesting that they may both contribute to the resistance phenotype of these isolates. The presence of both *mecA* and *mecC* in a single isolate is interesting, and suggests that the PBP2a proteins encoded by *mecA* and *mecC* might have distinct biological roles. This is further corroborated by the recent finding of a difference in temperature and substrate specificity of PBP2a encoded by *mecC* in comparison with *mecA.*^45^ It is of interest to find out how the two *mec* systems are regulated—whether regulation is hierarchal, with one system regulating the other, as seen with BlaR1/MecR1 regulation of *mecA*, and whether the recently described *mecR2* also regulates *mecC.*^46,47^ Understanding the regulation of *mecA* and *mecC* under different conditions might provide further insights into the biology of the two *mec* genes and identify suitable measures for reducing the selective pressures that maintain them.

We also identified that both the SCC*mec* type VII element and the SCC*mec*-*mecC* hybrid can excise from the chromosome and form a circular intermediate, despite the presence of SNPs in the *att*L2 and *att*R repeats. The fact that the C to A mutation in the *att*L2 does not prevent the excision reaction, as previously reported for the *att*B × *att*SCC integration reaction, suggests that this base is either not required for *att*L × *att*R recombination or that CcrA1 and CcrB5 have different sequence specificity compared with CcrA2 and CcrB2 from *S. aureus* N315 (71% and 86% amino acid identity, respectively). It is not possible to deduce if *mecA* and *mecC* were transferred together or independently into GVGS2 and GVGS3. There are no further regions of homology to either the SCC*mec* type XI or to the *mecC* region in *S. xylosus* S04009, which suggests that the *mecC* region was either transferred into the strain on a distinct element or has undergone significant decay. Recently, it was demonstrated that CcrA and CcrB recombinases can mediate recombination reactions between any combination of SCC*mec* repeats (*att*R/*att*L/*att*B/*att*SCC), raising the possibility that SCC*mec* type VII integrated into the *att*R of the *mecC* region or vice versa.^7^ The four genes immediately downstream from the *att*L2 of the *mecC* region are also present in the SCC*mec* VII element upstream and in the *S. fleurettii* chromosomal *mecA* locus, which has been suggested to be the template for the *mec* complex in *mecA* SCC*mec* elements (Figure 1).^12^ It is possible that these genes were also part of another SCC*mec* element that brought the *mecC* region into the chromosome. However, given that these genes are located outside of the *mecC* region *att*L2, it is equally likely that this just represents another, now decayed, SCC element present at the *orfX* locus.

The discrepancy of the presence of *erm*(C) and the lack of erythromycin resistance in GVGS2 is puzzling. The amino acid substitution in Erm(C) is unlikely to have caused a loss of function, as the Ile123Val mutation is present in a variable region of Erm-family proteins.^48,49^ A previous study has reported *S. aureus erm*(C)-positive isolates susceptible to erythromycin that could be selected to produce a resistance phenotype.^50^ Further investigation is required to understand the erythromycin-susceptible phenotype in GVGS2. In conclusion, this study further highlights that the *mecC* gene, like *mecA*, is disseminated widely amongst members of the *Staphylococcus* genus.

### Nucleotide accession numbers

The nucleotide sequences determined for GVGS2 were deposited in the EMBL database under accession number HG515014.

## Funding

This work was supported by a Medical Research Council Partnership Grant (G1001787/1) held between the Department of Veterinary Medicine, University of Cambridge (M. A. H.), the School of Clinical Medicine, University of Cambridge (S. J. P.), the Moredun Research Institute (R. N. Z.) and the Wellcome Trust Sanger Institute (J. P. and S. J. P.). S. J. P. receives support from the NIHR Cambridge Biomedical Research Centre. X. B. was supported by the China Scholarship Council and the Cambridge Overseas Trust. J. R. was supported by fellowship SFRH/BD/72675/2010 from Fundação para a Ciência e a Tecnologia.

## Transparency declarations

Competing interests: none to declare.

The funder had no role in the study design, data collection, analysis, decision to publish, or preparation of the manuscript.
